# Effect of propofol and remifentanil on cerebral perfusion and oxygenation in pigs: a systematic review

**DOI:** 10.1186/s13028-016-0223-6

**Published:** 2016-06-22

**Authors:** Mai Louise Grandsgaard Mikkelsen, Rikard Ambrus, James Edward Miles, Helle Harding Poulsen, Finn Borgbjerg Moltke, Thomas Eriksen

**Affiliations:** 1 Department of Veterinary Clinical and Animal Sciences, University of Copenhagen, 16 Dyrlægevej, 1870 Frederiksberg C, Denmark; 2 Department of Surgical Gastroenterology C, Rigshospitalet, University of Copenhagen, 9 Blegdamsvej, 2100 Copenhagen Ø, Denmark; 3 Department of Neuroanaesthesia, Rigshospitalet, University of Copenhagen, 9 Blegdamsvej, 2100 Copenhagen Ø, Denmark; 4 Department of Anaesthesia, Sealand Hospital, University of Copenhagen, 1 Lykkebækvej, 4600 Køge, Denmark

**Keywords:** Animal model, Brain, Oxygenation, Perfusion, Pig, Propofol, Remifentanil, Neuroanaesthesia

## Abstract

The objective of this review is to evaluate the existing literature with regard to the influence of propofol and remifentanil total intravenous anaesthesia (TIVA) on cerebral perfusion and oxygenation in healthy pigs. Anaesthesia has influence on cerebral haemodynamics and it is important not only in human but also in veterinary anaesthesia to preserve optimal regulation of cerebral haemodynamics. Propofol and remifentanil are widely used in neuroanaesthesia and are increasingly used in experimental animal studies. In translational models, the pig has advantages compared to small laboratory animals because of brain anatomy, metabolism, neurophysiological maturation, and cerebral haemodynamics. However, reported effects of propofol and remifentanil on cerebral perfusion and oxygenation in pigs have not been reviewed. An electronic search identified 99 articles in English. Title and abstract screening selected 29 articles for full-text evaluation of which 19 were excluded with reasons. Of the 10 peer-reviewed articles included for review, only three had propofol or remifentanil anaesthesia as the primary study objective and only two directly investigated the effect of anaesthesia on cerebral perfusion and oxygenation (CPO). The evidence evaluated in this systematic review is limited, not focused on propofol and remifentanil and possibly influenced by factors of potential importance for CPO assessment. In one study of healthy pigs, CPO measures were within normal ranges following propofol-remifentanil anaesthesia, and addition of a single remifentanil bolus did not affect regional cerebral oxygen saturation (rSO_2_). Even though the pool of evidence suggests that propofol and remifentanil alone or in combination have limited effects on CPO in healthy pigs, confirmative evidence is lacking.

## Background

Preservation of optimal cerebral perfusion and oxygenation (CPO) is the primary concern in assessing neurocritical patients undergoing surgery or sedation in the intensive care unit or for diagnostic procedures, and is equally important in both human and veterinary anaesthesia [1, 2]. Animal models have been used increasingly over the last decade to study the significance of influential factors on cerebral haemodynamics and to achieve better understanding of mechanisms regulating CPO. Rodents and primates are widely used in neuroscience but recently the interest for use of pigs in neuroscience has increased. The pig is readily available through commercial sources and the gyrencephalic porcine brain resembles the human brain in several areas of gross anatomy, growth, and development [3]. The pig brain shows similarities in gyral pattern, distribution of grey and white matter, cerebral blood flow, and metabolism to the human brain. In addition, similarities in neurophysiological development and post-natal maturation with human brains are of value in human neuro-embryology and paediatric neuro-science [3–5]. Various porcine models have been described with the objective of CPO evaluation, but mostly they have been developed in the context of neurological trauma or diseases, such as traumatic brain injury [6, 7], subdural haematoma [8], intracranial hypertension [9, 10], as well as epilepsy [11] and stroke [12].

Cerebral perfusion and cerebral oxygenation are two separate measures that are often used simultaneously and sometimes interchangeably as clinical assessment measures to evaluate the physiological status of the brain. Both measures are used to assess the risk of ischemic brain damage and thus to predict clinical outcome and prognosis of the patient. The cerebral perfusion is often clinically assessed by evaluation of changes in the cerebral blood flow (CBF). Cerebral blood flow is controlled by homeostatic regulation of the cerebral perfusion pressure (CPP) and cerebral vascular resistance (CVR). Mean arterial blood pressure (MAP) and the intracranial pressure (ICP) are determinants of CPP giving CPP = MAP−ICP, and CBF may therefore be assumed as CBF = MAP−ICP/CVR [13–16]. Overall, several physiological or pathological conditions therefore may influence cerebral perfusion. Physiological alterations in blood pressure, cerebral metabolic rate, temperature, arterial carbon dioxide or oxygen contents, blood viscosity and pathological conditions such as hypertension, vascular disease, trauma or seizures all may influence perfusion [2, 13–16]. Mean arterial pressure can under normal conditions, and for patients in supine position, adequately represent the CPP for evaluation of CBF [16]. Clinically, CPP is used as an indirect index for CBF [17]. The maintenance of constant and stable CBF is essential for optimal cerebral metabolism and function, and is under normal physiological conditions secured by intrinsic cerebral autoregulatory mechanisms [16]. Cerebral autoregulation is the haemodynamic ability of the cerebral vasculature to maintain a constant CBF despite changes in blood pressure and consequently changes in MAP [18]. The limit of this preservative regulatory mechanism is generally observed in the MAP range of 50–150 mmHg. When MAP is above or below these values, CBF varies markedly with MAP, because the ability of the individual vessels to change in diameter has been exhausted [19].

Cerebral oxygenation represents a measure for the amount of oxygen actually available for consumption and energy metabolism in the cerebral tissue, and can be defined as either the brain tissue partial pressure of oxygen (brPO_2_) [20], or as regional cerebral oxygen saturation (rSO_2_) [21]. Venous jugular oxygen saturation (SvjO_2_) normally provides an indirect measure for cerebral oxygen utilization, and thereby gives a qualitative assessment of CBF, but it is also used as a measure for global cerebral oxygen saturation. These parameters represent the oxygen content available within the tissue or in the vascular circuit, and are therefore dependent on CBF [1, 14, 22, 23].

Different methods and technologies have been established for the quantification and evaluation of cerebral haemodynamics, but no gold standard method has been fully validated to date, which makes comparison between the results of different studies challenging [1, 24, 25]. Historically, CBF has been studied by technically advanced methods like the nitrous oxide method [26], the krypton uptake method [27] or xenon 133 injection method [28, 29], but can be evaluated by modern techniques such as transcranial Doppler ultrasonography, laser Doppler flowmetry (LDF), positron emission tomography scanning, functional magnetic resonance imaging [14, 15, 20] or laser speckle contrast imaging [30, 31].

Target ranges for cerebral monitoring have been defined for healthy humans (Table 1), but are less well defined for pigs, which should be considered when assessing CPO in porcine models. CBF in pigs has been reported to decrease with age, being approximately 48, 44 and 27 ml/min/100 g in newborn, juvenile and adult pigs respectively [32], and thus may be comparable to the values identified in humans (Table 1).

**Table 1:** Target ranges for anaesthesia related cerebral perfusion and oxygenation measures in humans

Target	Measure	Limits	Reference
CerAutoReg^a^	mm Hg	50–150	[18]
MAP	mm Hg	>80	[18, 93]
CPP	mm Hg	50–70	[14, 17]
rSO_2_	%	60–80	[22, 90]
SvjO_2_	%	50–75	[15, 22]
brPO_2_	mm Hg	25–50	[17, 90]
CBF	ml/min/100 g	25–50	[94]
CarotidBF	ml/min	275 ± 52	[95]

*MAP*: mean arterial pressure; *ICP*: intracranial pressure; *CPP*: cerebral perfusion pressure; *rSO*_*2*_: regional cerebral oxygen saturation; *SvjO*_*2*_: jugular venous oxygen saturation; *brPO*_*2*_: brain tissue partial pressure of oxygen; *CBF*: cerebral blood flow; *CarotidBF*: carotid blood flow

^a^Upper and lower limits for cerebral autoregulation—highest in newborn lowest in adults

Normal target values for SvjO_2_ during anaesthesia have not been defined in pigs but Mutch et al. [33] and two studies included in this review reported mean values between 74.3 and 82.7 % (Table 6), and CPP has been found to vary with anaesthesia [34]. For brPO_2_, normal values in pigs have been found to be 25–30 mm Hg [35], which is similar to the range defined in humans (Table 1). Regional cerebral oxygen saturation measured by near infrared spectroscopy (NIRS) is simple, continuous and non-invasive and widely used in human neuroanaesthesia. Thus, it has been suggested that NIRS has potential as a central modality in the cerebral monitoring strategy [21]. NIRS has also been investigated in pigs and a normal value of 65 %, has been suggested [36], ranging from 57 to 72 %. In piglets, an ischaemic threshold of 35 % has also been suggested [37].

Most studies investigating CPO in porcine models are carried out in anaesthetized animals. General anaesthesia aims at depressing consciousness and cerebral activity. General anaesthesia influences systemic cardiovascular function and consequently will affect CPO. However, when properly monitored and managed, general anaesthesia should not alter CPO significantly in the healthy brain [38, 39]. The choice of anaesthesia should be evaluated when assessing studies evaluating CPO. Consideration should be given both to the isolated effect of single anaesthetic drugs, drug combinations as well as systemic physiologic status [39].

Inhalation anaesthesia elicits a dose-related suppressive effect of cerebral metabolism, but also has a direct dilating effect on the cerebral vessels that potentially will increase CBF. The net effect on CBF will be determined by dominating force of these competing and opposing effects [14, 40]. The general assumption is that most intravenous anaesthetic drugs will cause an indirect and dose-dependent decrease in CBF by decreasing cerebral metabolism [18, 41–44]. Only ketamine seems to be an exception to this, by expressing the opposite effect on cerebral metabolism, and thereby increasing CBF [43, 45]. Opioids in general are believed to have minimal effect on CBF in clinical doses, but their effect on cerebral haemodynamics may vary depending on patient status and anaesthetic drugs used [14].

The choice between inhalation and intravenous anaesthesia in neurosurgery is debated and even though no single anaesthetic drug or regimen appears absolutely superior to others [39], an increasing tendency to use intravenous protocols as the basic regimen for neurosurgical anaesthesia, has been reported [46–49]. Significant differences in baseline measurement of MAP and CPP between inhalation anaesthesia and total intravenous anaesthesia (TIVA) in piglets have been reported, but no differences in CBF or PbrO_2_ were however evident between the anaesthesia regimens [34].

Total intravenous anaesthesia (TIVA) with a combination of propofol and remifentanil is an anaesthetic regimen that is widely used in neuroanaesthesia, and has advantages over inhalation anaesthesia (sevoflurane and isoflurane) with regard to desirable traits like fast recovery, a low incidence of nausea and vomiting, anticonvulsive potential, preservation of cerebral autoregulation and neuroprotection [40, 49–51]. Propofol is the most widely used intravenous anaesthetic agent in both human and veterinary medicine [49, 52–54]. Due to its global depressant properties in the central nervous system, it acts as a very potent hypnotic agent, which can be used for both induction and maintenance of general anaesthesia, and for sedation. Its favourable pharmacokinetic profile ensures a fast onset of action and rapid recovery even after prolonged administration [55]. Propofol may indirectly reduce CBF mainly due to depression of the systemic blood pressure [53, 55]. The hypotensive effect is caused by a decrease in sympathetic activity and intracellular calcium flux resulting in vasodilation [55]. In pigs, the vasodilation caused by propofol has also been related to release of nitric oxide by the vascular endothelium [55]. Propofol has little or no effect on the conductive system of the heart and does not cause myocardial depression, but inhibits the tachycardiac response to hypotension by inhibition of the baroreceptor reflex [53, 55]. The overall cerebral effect of propofol has been demonstrated to reduce CBF, cerebral metabolic rate of oxygen and intracranial pressure, but it also acts as an antioxidant and has neuroprotective properties [53, 56, 57]. Remifentanil is an ultra-short-acting opioid with very potent and selective µ-receptor agonist activity. It is preferred to fentanyl or other opioids due to its non-organ specific metabolism by blood and tissue esterases, which consequently results in a faster clearance and thereby faster recovery independent of infusion duration [58]. The main cardiovascular impact of remifentanil is dominated by moderate bradycardia and a marked decrease in systemic blood pressure. The use of remifentanil in humans thus, has an increased incidence of hypotensive periods in comparison with other opioids [58, 59]. In contrast to this and compared to fentanyl-sevoflurane, remifentanil-propofol anaesthesia is in human clinical studies also reported to increase the incidence of arterial hypertension, hence the influence of remifentanil on CPO is somewhat unpredictable and may depend on the anaesthetic agent used [59]. In humans low and moderate dose remifentanil caused increase in regional CBF while remifentanil in high, supra-clinical doses caused a decrease in regional CBF [59], and no impairment of the cerebrovascular carbon dioxide reactivity [60]. It has been suggested that remifentanil in general decreases the cerebral metabolic rate of oxygen and only has minimal effect on intra cranial pressure [59, 61] and that the combined use of propofol and remifentanil preserves and improves cerebral autoregulation [59]. The overall effects of propofol and remifentanil on CBF and cerebral autoregulation in selected animal species are summarised in Table 2. Since the pig is increasingly of interest as a translational model in neurocritical research, it is important to reveal whether propofol and remifentanil influence CPO differently in pigs compared to humans. The objective of this review is to evaluate the existing literature with regard to the influence of propofol and remifentanil TIVA on cerebral perfusion and oxygenation in healthy pigs.

**Table 2:** Summary of the effects of propofol and remifentanil on cerebral blood flow and cerebral autoregulation in selected animal species used in experimental neuroscience

Species	CBF			CA		References
	P	R	PR	P	PR	
Humans	↓	↑↓*	↓	Preserved	Preserved	[18, 52, 55, 56, 58]
Dog	↓	↓	•	Preserved	•	[96, 97]
Rabbit	↓	•	•	•	•	[98]
Rat	↓	•	•	Preserved	•	[99]

*CBF*: cerebral blood flow; *CA*: cerebral autoregulation; *P*: propofol; *R*: remifentanil; *PR*: propofol-remifentanil combination; ↑: increased; ↓: decreased; •: uncertain; *: dose dependant

## Search strategy

On May 10th 2016, an electronic database search was made in Agricola 1970 to April 2016, Agris 1975 to March 2016, Biosis Previews 1969–2009, CAB Abstracts 1910–2016 week 17, Embase 1974–2016 May 9th, International Pharmaceutical Abstracts 1970–April 2016, Zoological Record 1978–2009, Epub ahead of print, In-process and Other non-indexed citations, MEDLINE daily and MEDLINE 1946 to present. The specific terms used for the different search components were: (1) cerebral or brain or central nervous system (CNS), (2) CNS, (3) 1 OR 2, (4) pig or pigs or piglet or piglets or swine or swines or landrace or porcine or minipig or minipigs, (5) 3 and 4, (6) propofol or remifentanil or TIVA, (7) TIVA, (8) TIVA, (9) 6 or 7 or 8, (10) 5 and 9, (11) guinea or guineapig or guineapigs or guinea-pig or guinea-pigs, (12) 10 not 11. The only filter applied was English, and the reference lists of relevant articles and recent reviews were hand-searched for additional studies. Three authors did the screening individually and any disagreement in selection were settled by mutual agreement.

**Fig. 1:**
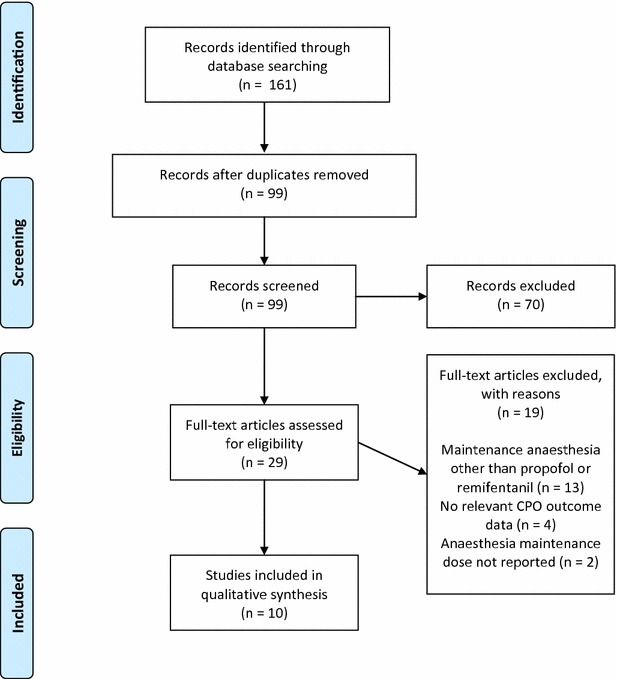
PRISMA flow chart, Based on [102]

Studies that evaluated CPO and used propofol or remifentanil for TIVA in healthy pigs of all ages and breeds were included in the systematic review. This also included studies that only indirectly assessed the effect of propofol or remifentanil on CPO. Studies where additional anaesthetic drugs were given for maintenance of anaesthesia were excluded. Eligible records (Fig. 1) were retrieved in full text and screened for relevant anaesthesia protocols and outcome data.

## Data extraction

This systematic review is based on the principles recommended by the SYRCLE collaboration [62] and O´Connors and Sargeant [63]. Primary outcome data relevant to this systematic review were any CPO parameter. Secondary outcome data extracted were publication year, study type, number of animals, breed, bodyweight, age, gender, doses of preanaesthetic and other medications, doses of propofol and remifentanil, and MAP.

Synthesis of the CPO-data was made on the basis of means, standard deviations and 95 % confidence intervals and is discussed with regard to secondary outcome data. Standard errors of means were transformed to standard deviations. If not reported, animal age was estimated based on weight [64–66].

## Review

### Search results

The electronic search identified 99 articles in english, of which 29 were eligible after title and abstract screening. Of these, 19 studies were excluded after full text examination (Fig. 1). Of the10 peer-reviewed articles included in this review, only three had propofol or remifentanil anaesthesia as the primary study objective, and only two investigated the effect of anaesthesia on CPO (Table 3). The studies were published between 2001 and 2014, and all but one, were non-survival studies. Post-intervention data from the survival study were not included in this review.

**Tabel 3:** Anaesthesia relevant data

Study ID [reference]	Premedication. drug (dose^a^)	Propofol dose^b^ (induction/maintenance)	Remifentanil dose
Boezaart [68]	Ketamine (10 mg/kg im) Pancuronium (0.1 mg/kg)	2.0 mg/kg/3.0 mg/kg/h	0.35 mikrogram/kg/min
Kajimoto et al. [82]	Ketamine (33 mg/kg im) xylazine (2 mg/kg im)	1.0–2.0 mg/kg/15.0 mg/kg/h for 15 min then 12.0/mg/h for 45 min then 9.0 mg/kg/h for 60 min then 7.5 mg/kg for 120 min	Not used
Lurie et al. [73]	Ketamine (21.9–25.0 mg/kg im)	2.3 mg/kg + 1.0 mg/kg/9.6 mg/kg/h	Not used
Navarro et al. [70]	Ketamine/Telazol/xylazine im (not reported)	1.5 mg/kg/9.0 mg/kg/h	Not used
Silva et al. [71]	Azaperone (4 mg/kg im)	4.0 mg/kg/15.0 mg/kg/h	0.3 mikrogram/kg/min followed by 0.2 mikrogram/kg/min
Silva et al. [67]	Azaperone (4 mg/kg im)	4.0 mg/kg/15.0 mg/kg/h	0.3 mikrogram/kg/min + 5 mikrogram/kg bolus
Srinivasan et al. [72]	Ketamine (21.2–31.3 mg/kg im)^c^	2.0–3.0 mg/kg/9.6 mg/kg	Not used
Yannopoulos et al. [100]	Ketamine (23.0–23.7 mg/kg im)	1.0 mg/kg/9.6 mg/kg/h	Not used
Yannopoulos et al. [101]	Ketamine (21.7–23.5 mg/kg im)	2.0 mg/kg/9.6 mg/kg/h	Not used
Yannopoulos et al. [69]	Ketamine (25.6–30.3 mg/kg im)	2.3 mg/kg/9.6 mg/kg/h	Not used

^a^Doses are calculated based on animal weight

^b^Doses reported as mikrogram/kg/min are converted to mg/kg/h for easier comparison

^c^Calculated under the assumption that lowest dose was given to animals with lowest weight

#### Animals

Data from a total of 133 animals are included in this review. All studies used farm pigs. Eight of the studies used animals with a bodyweight between 16 and 33 kg, while one used animals between 7.5 and 14.5 kg and another between 21.5 and 92.8 kg. Seven studies used female and three used male pigs: none of the studies used both (Table 4).

**Tabel 4:** Animal and publication relevant data

Study ID [reference]	Animal intervention study	Random sequence allocation	n	Gender/pig breed	Weight (kg)^a^	Age in weeks
Boezaart [68]	1 group	NA	10	Female/pigs	22–28	8–9^b^
Kajimoto et al. [82]	2 groups	No	14	Male/yorkshire	7.8–14.5	4–6^c^
Lurie et al. [73]	2 groups	Yes	22	Female/farm pigs	30.4 ± 1.3	9–11^b^
Navarro et al. [70]	1 group	NA	6	Female/swine	21.5–92.8	8–21^b^
Silva et al. [71]	2 groups	Yes	12	Male/large white	27.0 ± 3.6	12^d^
Silva et al. [67]	1 group	NA	12	Male/Large white	26.2 ± 3.6	12^d^
Srinivasan et al. [72]	1 group	NA	13	Female/domestic farm pigs	16–33	12–16
Yannopoulos et al. [100]	2 groups	Yes	16	Female/farm pigs	30 ± 0.5	10–11^b^
Yannopoulos et al. [101]	2 groups	No	12	Female/yorkshire-farm cross	31 ± 1.2	10–11^b^
Yannopoulos et al. [69]	2 groups	Yes	16	Female/farm pigs	25.2 ± 2.1	8–9^b^

*n*: number of animals; *NA*: not applicable

^a^Mean ± SD or upper and lower limits

^b^Age in weeks estimated from weight post hoc if not reported

^c^Reported as 27–41 days

^d^Reported as 3 months

#### Anaesthesia

Three studies used a combination of propofol and remifentanil, and seven studies used propofol alone (Table 3). In all studies, anaesthesia was induced with propofol intravenously (iv) (1–4 mg/kg) and maintained with propofol (3–15 mg/kg/h). In 3 studies remifentanil was given in a dose range of 0.2–0.35 µg/kg/min. In addition to propofol and remifentanil infusion, Silva et al. [67] investigated the effect of a single intravenous bolus of 5 µg/kg remifentanil. Intramuscularly (im) azaperone 4 mg/kg was used in two studies while ketamine, 10–33 mg/kg im, was used for premedication in eight of the studies. Six of these studies used ketamine alone, one added xylazine and one added xylazine, tiletamine and zolazepam. Single bolus pancuronium was used for muscle relaxation in one study [68], and flunixin, 2.2 mg/kg im was used pre-emptively for postoperative analgesia in one single survival study [69].

#### Study design

The included studies were animal intervention studies. Four studies used a 1-group design with pre-post-test assessment. Six 6 studies used a 2-group design of which 4 used random sequence allocation. Only Silva et al. [67] studied the effects of propofol-remifentanil anaesthesia on CPO as the primary objective. The remaining 9 studies did not have CPO in relation to anaesthesia as their primary objective. However, all were clear in their description of objectives and possible factors of influence are discussed below.

### Extracted data

Predefined respiratory baseline limits were similar across studies, with target limits set at a respiratory rate of 10–14/min, tidal volume of 10–20 ml/kg, arterial CO_2_ (PaCO_2_) or end tidal CO_2_ (EtCO_2_) between 35 and 45 mmHg, arterial oxygen (PaO_2_) >80 mm Hg and oxygen saturation (SpO_2_) >90 %. However, not all parameters were reported in all studies. Predefined haemodynamic baseline limits were not reported in detail in any of the studies, but Boezaart [68], Navarro et al. [70], Silva et al. [71] and Srinivasan et al. [72], reported that haemodynamic baseline recordings had to be stable prior to intervention. MAP and the 95 % confidence interval of all studies were within the limits of cerebral autoregulation (Table 5).

**Table 5:** Means and 95 % confidence intervals of reported MAP

Study ID [reference]	n	Outcome	Measure	Mean	CI 95 % (LL)	CI 95 % (UL)
Boezaart [68]	10	MAP	mm Hg	89.0	82.6	95.4
Kajimoto et al. [82]	7	MAP	mm Hg	69.0	63.1	74.9
Lurie et al. [73]	22	MAP	mm Hg	91.8	85.3	98.2
Navarro et al. [70]	6	MAP	mm Hg	Range	70.0	110.0
Silva et al. [67]	12	MAP	mm Hg	72.7	67.4	78.0
Silva et al. [71]	12	MAP	mm Hg	73.0	67.3	78.7
Yannopoulos et al. [100]	16	MAP	mm Hg	81.0	73.9	88.1
Yannopoulos et al. [101]	12	MAP	mm Hg	90.0	81.2	98.8
Yannopoulos et al. [69]	16	MAP^a^	mm Hg	90.1	83.8	96.4

*n*: number of animals; *MAP*: mean arterial pressure; *CI*: confidence interval; *LL*: lower limit; *UL*: upper limit

^a^Calculated from systolic and diastolic blood pressure

Cerebral blood flow was reported on the basis of directly measured transdural flow in tissue perfusion units, indirectly measured carotid artery blood flow in ml/min or jugular venous oxygen saturation (SvjO_2_) in  % or as cerebral perfusion pressure (CPP) in mm Hg. Mean CPP was reported in four studies and ranged from 68 to 74.8 mm Hg (Table 6).

**Table 6:** Means and 95 % confidence levels of reported cerebral perfusion measures

Study ID [reference]	n	Outcome	Measure	Mean	CI 95 % (LL)	CI 95 % (UL)
Boezaart [68]	10	CBF	TPU	44.7	29.2	60.2
Lurie et al. [73]	22	CarotidBF	ml/min	119.4	101.8	136.9
Silva et al. [67]	12	SvjO2	%	82.7	77.0	88.3
Silva et al. [71]	12	SvjO2	%	79.0	73.9	84.1
Srinivasan et al. [72]	13	CPP	mm Hg	73.6	67.0	80.2
Yannopoulos et al. [69]	16	CarotidBF	ml/min	174.8	153.6	196.0
Yannopoulos et al. [100]	16	CPP	mm Hg	68.0	61.6	74.4
Yannopoulos et al. [101]	12	CPP	mm Hg	74.0	64.6	83.4
Yannopoulos et al. [69]	16	CPP	mm Hg	74.8	67.2	82.3

*n*: number of animals; *CI*: confidence interval; *LL*: lower limit; *UL*: upper limit; *CBF*: cerebral blood blow; *CarotidBF*: carotid blood flow; *SvjO2*: jugular venous haemoglobinoxygen saturation; *CPP*: cerebral perfusion pressure

Cerebral oxygenation was reported on the basis of direct measurement of brain tissue partial pressure of oxygen (brPO_2_) in mm Hg or indirect transcranial measurement of regional cerebral oxygen saturation (rSO_2_) in %. Data for brPO_2_ from one study [73] was not reported in absolute values, but were extracted from a graph. Values for rSO_2_ between 51 and 65 % were reported in three studies while one study reported an rSO_2_ range from 65 to 80 % (Table 7).

**Table 7:** Means and 95 % confidence intervals of reported cerebral oxygenation measures

Study ID [reference]	n	Outcome	Measure	Mean	CI 95 % (LL)	CI 95 % (UL)
Kajimoto et al. [82]	7	rSO2	%	51.0	47.1	54.9
Lurie et al. [73]	22	brPO2^a^	mm Hg	13.2	9.9	16.5
Navarro et al. [70]	6	rSO2	%	Range	65.0	80.0
Silva et al. [67]	12	rSO2	%	62.3	58.6	65.9
Silva et al. [71]	12	rSO2	%	65.0	61.6	68.4

*n*: number of animals; *CI*: confidence interval; *LL*: lower limit; *UL*: upper limit; *rSO2*: regional cerebral oxygene saturation; *brPO2*: brain tissue partial pressure of oxygen

^a^Extrapolated from graph

### Evaluation of the effects of propofol and remifentanil

This systematic review was not able to identify studies with the primary objective to investigate the combined effects of propofol and remifentanil TIVA on cerebral perfusion and oxygenation in pigs. Thus, the question of how propofol and remifentanil affects CPO in healthy pigs remains uncertain. Silva et al. [67] investigated the effect of a single high dose remifentanil bolus (5 µg/kg iv) on cerebral oxygenation during TIVA with propofol and remifentanil. They reported CPO outcome measures during propofol-remifentanil infusion (Tables 5, 6, 7), that were within the normal target limits, while a single bolus of remifentanil significantly lowered cerebral oxygenation, yet still within normal limits (Table 1). Silva et al. [67] suggest that this decrease may be associated with a decrease in cardiac output rather than altered cerebral metabolism. The primary aim of the remaining nine articles was not to investigate the effects of propofol or propofol-remifentanil anaesthesia on CPO. Hence, data from these studies may have been influenced by factors other than anaesthesia with propofol or remifentanil. Beydon et al. [74] who studied bispectral index stability in response to haemodynamic instability, suggested that a propofol-remifentanil TIVA with a lower dose propofol (8.4 mg/kg/h) and a higher dose of remifentanil (0.54 µg/kg/min) provides a more stable anaesthetic depth compared to propofol-remifentanil TIVA with a higher dose of propofol (26.7 mg/kg/h) and lower dose of remifentanil (0.34 µg/kg/min). Relative to the findings made by Beydon et al. [74], Boezaart et al. [68] used a very low dose of propofol for maintenance (3 mg/kg/h) and a low dose of remifentanil, while the remainder of articles reviewed here, used a low–mid range propofol dose and a low remifentanil dose (Table 3). Silva et al. [71] defined a stable level of anaesthesia as total muscle relaxation, absence of palpebral reflex, and absence of hemodynamic response to interdigital space clamping. Srinivasan et al. [72] titrated anaesthesia on the basis of heart rate, blood pressure, tail and hoof response and spontaneous breathing. The depth of anaesthesia and consequently the stress response to surgical stimulation thus may differ between studies, which makes comparison difficult. None of the studies however, reported concerns about insufficient anaesthetic depth.

#### Choice of model

The studies included used farm pigs of different breeds that were only specified in 4 of the studies (Table 4). Farm pig breeding standards vary from country to country resulting in pig breeds of varying anatomy and physiology. When used in scientific studies thus, detailed consideration should be given to the choice of pig breed [3, 75]. None of the studies reviewed here used mini or micro pig breeds but different farm pig breeds only, hence we find it acceptable to consider results as relatively comparable provided that age is taken into consideration. Based on the relationship between bodyweight and age, the animals included were approximately between 1 and 6 months old [64, 65]. For neurodevelopmental comparison to the human brain, it has been estimated that 1 week of life in the piglet approximates 1 month of life in humans [66]. Therefore, these animals could be considered equivalent to infants between 4 months and 2 years of age and may be the basis for a translational model of this paediatric subpopulation, while it seems less advisable to use this model in studies aiming at the human adult or geriatric subpopulation [76]. Cerebral autoregulation has been shown to be impaired more extensively in newborn than in juvenile pigs in response to traumatic brain injury [4], which indicates an age-dependent maturation of the cerebral autoregulatory response. Consequently, the youngest animals would be more sensitive to periods of hypotension (e.g. in response to anaesthetic induction) and potentially result in an over-interpretation of differing CPO responses between animals at both ends of the age span. This may explain the relatively broad range of rSO_2_ values in the study by Navarro et al. [70], since this study was conducted on few animals (n = 6) with body weights ranging from 21.5 to 92.8 kg and estimated ages of approximately 8–21 weeks. In parallel, the relatively wide range in MAP observed may also be a result of an uneven sensitivity to the propofol doses between animals with different age. As a result, an inter-animal comparison could be biased due to unequal physiological prerequisites, even if cerebral oxygenation was still within target limits for pigs.

Gender has been described as a factor influencing cerebral autoregulation in piglets [77, 78]. None of the selected studies discussed the choice of animal gender.

#### Anaesthesia and drug contamination

Anaesthesia related discussion was brief and not given in all of the selected publications and main motivators for the choice of anaesthesia were animal welfare and analgesia while less attention was given to the potential influence of anaesthesia on their main outcome measures. Premedication was an integrated part of the anaesthetic protocol in all 10 studies and might influence outcome data, possibly in a dose dependent manner. Ketamine, which was used in eight out of the ten studies included in this review, is recommended for premedication-induction and reduction of stress response in pigs [39]. Ketamine has also been reported to increase CBF, ICP, and cerebral metabolic rate at high doses in pigs [40, 45, 79–81]. None of the studies addressed the risk of drug contamination bias or length of washout periods, even though doses ≥30 mg/kg of ketamine were used for premedication in some of the studies [69, 72, 82]. However, from the trial descriptions we believe that the preparatory periods were long enough to reduce the risk of drug contamination bias in the assessed studies [83]. If this also is the case for the two studies [70, 82] that combined ketamine premedication with either xylazine alone or ketamine with tiletamine, zolazepam and xylazine, is unclear. However another α_2_-agonist, medetomidine, has been reported to have a more prolonged effect on heart rate in minipigs [84] and xylazine has been reported to reduce local cerebral CBF in rats [85]. Azaperone, which was used for sedation in two studies (Table 3) has been reported to influence both cerebral and systemic haemodynamics, but is in general believed to have little influence on haemodynamics [86].

Boezaart [68] used pancuronium for muscle relaxation. High-dose pancuronium has been reported to widen the cerebral autoregulatory range in newborn piglets, but the single bolus of 0.1 mg/kg, which was given at time of premedication is not expected to have influenced cerebral haemodynamics [87].

In one survival study [69] the nonsteroidal anti-inflammatory drug flunixin was given for post-operative analgesia intramuscularly, 1 h before surgical intervention. Flunixin has a relatively fast onset, and a long half-life of approximately 7 h in pigs [88]. The acute effect of flunixin on the haemodynamics of the normal pig brain has not been investigated, but other cyclooxygenase inhibitors have been reported to affect CBF when administered intravenously. Prostaglandin synthesis is cyclooxygenase dependent and plays a central role in maintaining CBF and cerebral autoregulation. It may be speculated that inhibition of prostaglandin synthesis by flunixin may influence CBF and consequently cerebral oxygenation [89]. The potential influence of flunixin on CPO assessment was not discussed by Yannopoulos et al. [69].

Premedication, in terms of dose, time from administration to the beginning of CPO measurement, and expected serum concentration of drugs and active metabolites is relevant to assessing the risk of drug related outcome contamination. Even though this is considered insignificant for primary outcome assessment, this was not addressed in any of the 10 articles reviewed.

#### Methods of CPO measurements

Monitoring CPO is a complex assessment of principally different variables such as systemic blood pressure, intra cranial pressure, tissue oxygen tension or haemoglobin saturation—all of which may be influenced by choice of monitoring technique [1, 20, 31, 90].

It is difficult to compare the cerebral perfusion measures reported in the selected articles. CBF was reported in 4 studies (Table 6). Lurie et al. [73] and Yannopoulos et al. [69] quantified a global measure of CBF by identical techniques of LDF over the exposed left common carotid artery. Lurie et al. [73] reported significantly lower values than Yannopoulos et al. [69] despite similar animal weight, gender and anaesthetic protocols. Yannopoulos et al. [69] additionally used 2.2 mg/kg flunixin im prior to surgery, which would be expected to reveal the opposite relationship, since flunixin in theory may have a CBF lowering effect. However, wide carotid blood flow limits have been reported earlier. Boezaart [68] also used LDF, but measured CBF locally through a drilled craniotomy, and reported it as relative tissue perfusion units, where the other studies reported it as absolute ml/min. Comparison of the CBF from the three studies is difficult because of the difference in measuring techniques, brain area of interest and wide normal flow limits [32, 91].

CPP was reported in four studies with a range between 68 and 74.8 mmHg, which is similar to previously reported CPP in healthy pigs anaesthetized with midazolam and fentanyl after ketamine and xylazine sedation [34]. More studies are required to verify this, and to determine a more exact range for CPP in pigs during different types of anaesthesia. Collectively however, none of the reported cerebral perfusion measures (Table 6) were outside the normal ranges for humans that are available in the literature (Table 1).

Cerebral oxygenation is a measure of oxygen available for consumption and energy metabolism in the brain. Lurie et al. [73] reported a mean brPO_2_ baseline value of 13.2 mm Hg, 95 % CI [9.9–16.5 mm Hg]. This brPO_2_ is well outside the target ranges available in healthy swine of 25–30 mm Hg [35] and further, is within the ischaemic range of <15 mm Hg in humans [1, 22, 92]. Lurie et al. [73] used the baseline as a relative comparison to post-interventional measures, however the relatively low brPO_2_ is not discussed in the article.

Non-invasive measurement of rSO_2_ with near infrared spectroscopy was performed in 4 studies and baseline values were reported for both propofol studies and for propofol and remifentanil studies (Table 7). Navarro et al. [70] reported an rSO_2_ range between 65 and 80 % with the upper range slightly above the target range of 57–72 % [36]. Kajimoto et al. [82] reported an rSO_2_ mean of 51 %, 95 % CI (47.1; 54.9). This lower oxygenation measure could be related to the use of younger animals in this study and the high-dose ketamine and xylazine in the premedication used in this experiment. The earlier stage of neurodevelopment could render the animals more susceptible to sedative agents, which would lead to a more extensive metabolic cerebral depression and therefore lower oxygen demand.

## Conclusions

Even though the very limited pool of evidence reviewed here, it suggests that propofol and remifentanil alone or in combination have limited effects on CPO in healthy pigs, confirmative evidence is lacking. In one study baseline CPO measures were within normal target ranges in healthy pigs during propofol-remifentanil TIVA. Addition of a single remifentanil dose significantly reduced rSO_2_ but not beyond normal target ranges.

There is a need for standardised porcine studies with the primary objective of studying the influence of anaesthetic drugs on CPO in non-diseased animals.

## Abbreviations

brPO_2_: brain tissue partial pressure of oxygen
CBF: cerebral blood flow
CPO: cerebral perfusion and oxygenation
CPP: cerebral perfusion pressure
LDF: laser Doppler flow
MAP: mean arterial pressure
rSO_2_: regional cerebral oxygen saturation
SvjO_2_: jugular venous oxygen saturation
TIVA: total intravenous anaesthesia
